# Computerized Testing Software for Assessing Interference Suppression in Children and Adults: The Bivalent Shape Task (BST)

**DOI:** 10.5334/jors.ak

**Published:** 2014-05-23

**Authors:** Shane T. Mueller, Alena G. Esposito

**Affiliations:** 1 Asst. Professor, Dept. of Cognitive and Learning Sciences, Michigan Technological University, Michigan, Hoguhton, United States; 2 North Carolina State University, Department of Psychology, Raleigh, North Carolina, United States

**Keywords:** computerized cognitive testing, inhibition, interference suppression, Stroop interference, children and adults, non-linguistic interference test

## Abstract

We describe the Bivalent Shape Task (BST), software using the Psychology Experiment Building Language (PEBL), for testing of cognitive interference and the ability to suppress interference. The test is available via the GNU Public License, Version 3 (GPLv3), is freely modifiable, and has been tested on both children and adults and found to provide a simple and fast non-verbal measure of cognitive interference and suppression that requires no reading.

## (1) Overview

### Introduction

Research on bilingualism in children has suggested that children who are bilingual develop improved ability to suppress irrelevant information, and that this advantage may transfer to non-linguistic contexts. To examine this, a computerized cognitive test called the Bivalent Shape Task (BST) was recently published by Esposito and Colleagues [1,2], implemented using the Psychology Experiment Building Language (PEBL; see [3,4,5,6]). The task is similar at a high level to a number of traditional attentional interference tests (e.g., Dimensional Change Card Sort [7], the color-word Stroop test [8], the Simon interference task [9], and Eriksen's flanker task [10]), but introduces new aspects that make it appropriate for testing children and adults in a non-linguistic setting. In addition, the task can be used across the lifespan in developmental studies. Furthermore, the software includes the ability to examine a number of related stimulus-response conditions that may be of interest to future researchers.

**Fig. 1:**
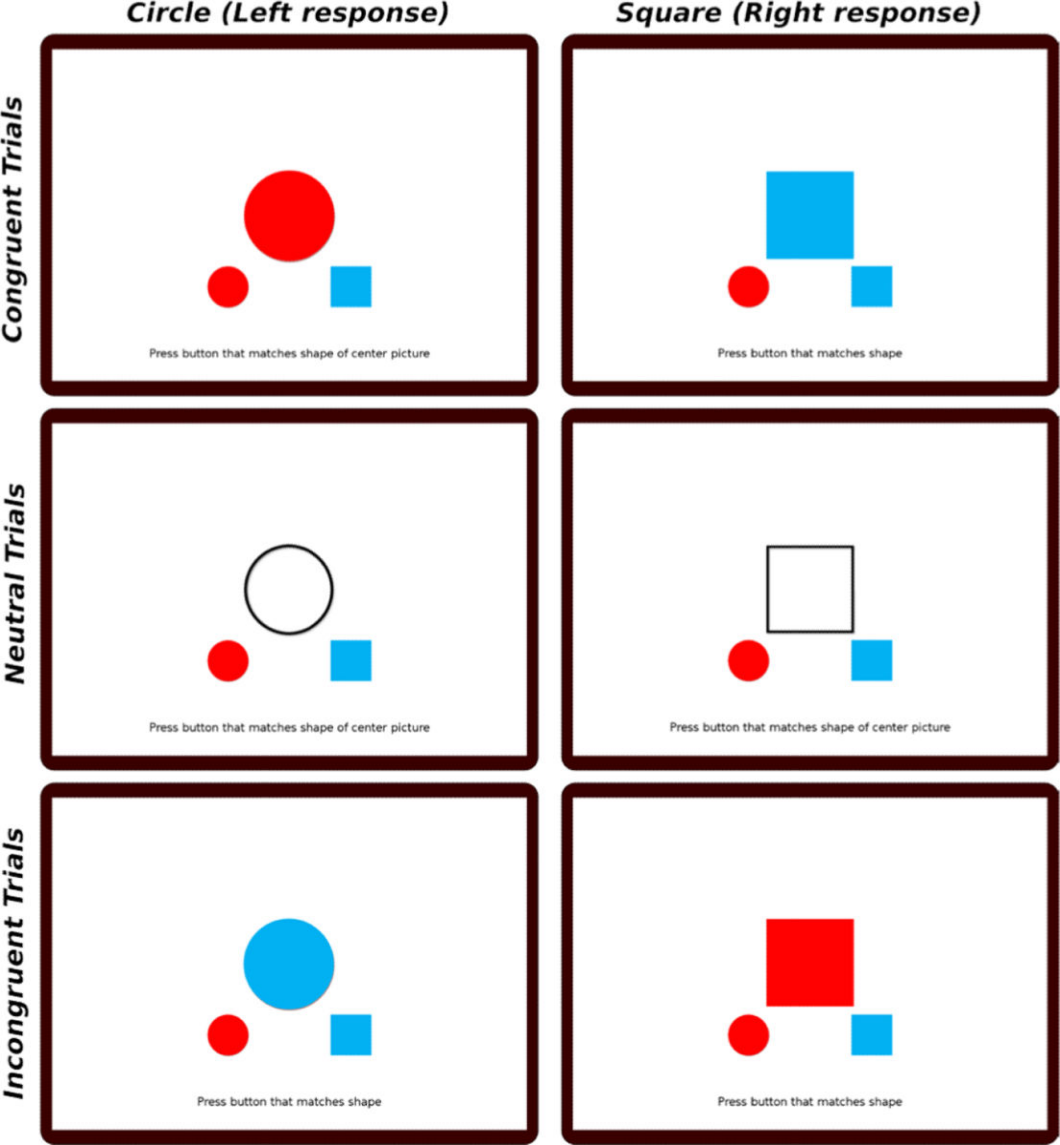
The six trial types in the BST for which the participant must sort the shape, but not the color, of the stimulus. Rows show congruent, neutral and incongruent trials; columns show circle versus square stimuli.

The basic logic of the test requires the participant to determine whether a shape at the center of the screen is a circle or a square. Circles are always responded to with the left response, and squares are always responded to with the right response (depending on the response configuration, either via the keyboard, clicking with a mouse, or touching the screen). Visual response cues are provided below the stimulus, indicating the side of the response. However, these response cues are shaded in either red or blue. In all cases, color is irrelevant and not used to make the decision. The stimulus shape is presented either in red, blue, or an unfilled black outline. Thus, three basic trial types exist: congruent trials, in which the irrelevant color of the stimulus matches the response cue; neutral, in which the stimulus is black and white, and incongruent, in which the (irrelevant) color mismatches the response cue. Dependent measures of interest are the speed and accuracy with which participants are able to make the decision. These trial types, for both circle and square stimuli, are shown in **Figure 1**.

The software is available as part of the PEBL Test Battery, and will be included as in Version 0.14 of PEBL. To run the BST, one first must install the PEBL system, precompiled versions of which are available for Microsoft Windows, OS X, and Linux (using a .deb package) at http://pebl.sourceforge.net. PEBL is installed by either running the setup executable file (on Microsoft Windows) or dragging the uncompressed PEBL application bundle into the Applications folder (on Apple's OS X). This typically requires administrator access. After PEBL is installed, the first time it is run by a user, a set of testing files will be automatically copied to a folder within the user's “Documents” folder, called pebl-exp-XX, where XX indicates the version of PEBL being installed. That is, for Version 0.13, the destination will be Documents\pebl-exp-0.13. For PEBL Version 0.14 and later, files related to the BST task will be within the battery\BST subfolder of that folder. Those using PEBL 0.13 will need to download and install the BST separately after PEBL is installed. To do so, the compressed archive must be downloaded from one of the locations indicated in the Availability section of this paper. The compressed folder can be uncompressed anywhere, but we recommend placing it within the Documents\pebl-exp-XX\battery folder just described to allow it to be accessed via the PEBL launcher, and the instructions below assume that it is placed in this location.

**Fig. 2:**
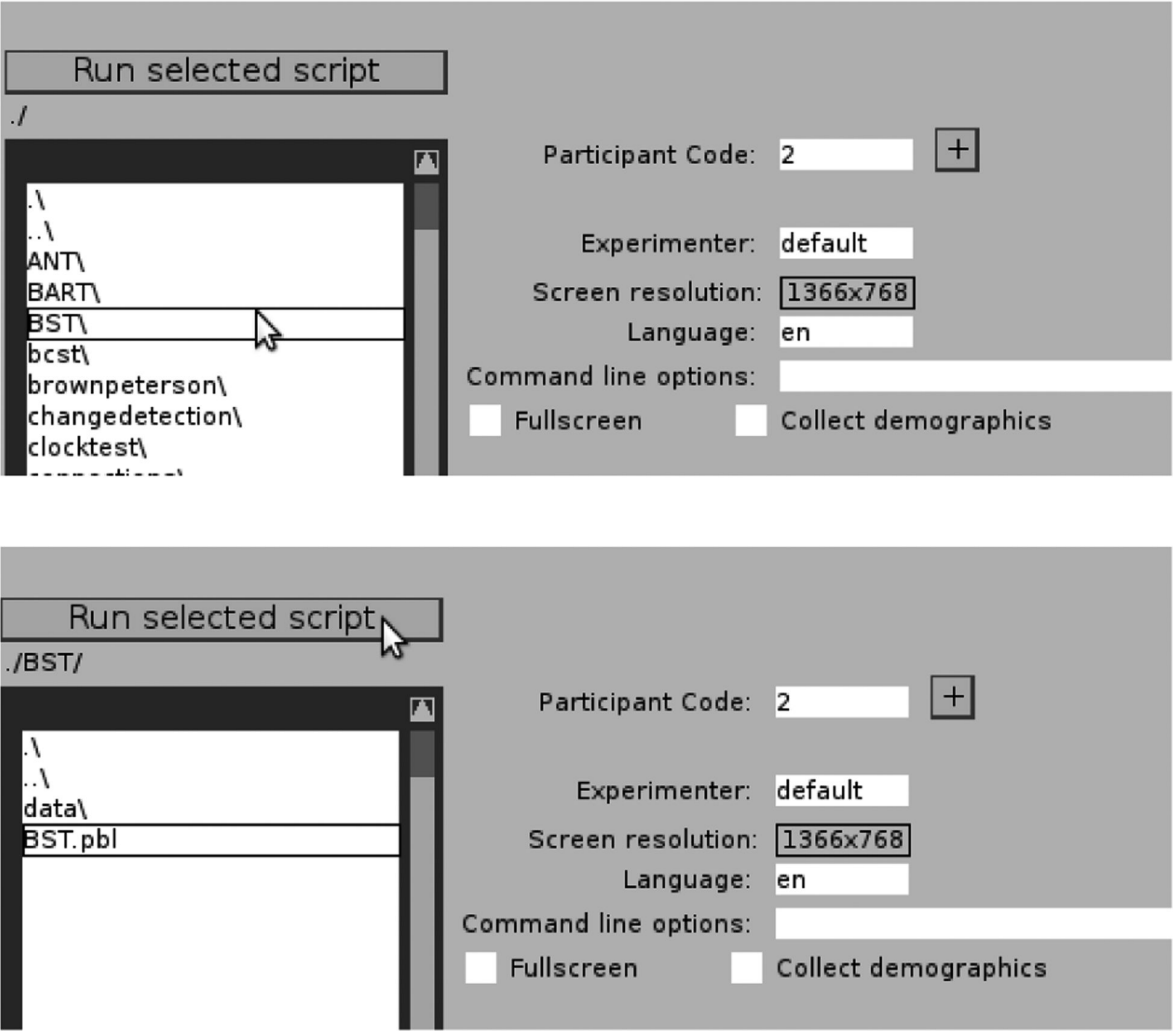
Depiction of the steps used to run the BST via the PEBL Launcher. First (top panel) the BST folder must be selected and opened by clicking twice from within the battery\ folder. Next (bottom panel), the BST.pbl file must be selected, and the test launched using the ‘Run selected script’ button. Other experimental parameters, such as participant code, screen resolution, etc. can be controlled via the launcher.

To run the BST task, the PEBL launcher must first be started. After the first time PEBL is run (during which local installation is performed), the launcher will run whenever the PEBL executable icon is used. The launcher allows PEBL tests to be browsed, selected, and run, and allows a number of functions for managing experiments (e.g., recording participant codes, setting up series of tests to complete, and selecting the testing language, and other parameters). A series of directories are displayed on the left panel, each one of which can be opened by clicking twice on the name. The Launcher starts in the pebl-exp-XX\ folder, and so to launch the BST, first click twice on the battery\ folder, and then twice on the BST folder, as shown in **Figure 2**. The BST.pbl file will be selected by default, and the test can be launched by clicking the ‘Run selected script’ button. Once completed, the data from the task will be saved in the data\ folder within the BST folder. Details regarding the data format are found in the next section.

### Implementation/architecture

The software was implemented in the Psychology Experiment Building Language [3,4,5,6]. PEBL is a cross-platform open source programming language designed to implement both simple and complex psychological tests. PEBL is programmed with a text-based syntax that is both structured and forgiving, enabling novice programmers to edit tests and create new tests. The basic logic of the BST is as follows:

- **Basic instructions.** Written instructions appear on the screen at the beginning of the test. When being taken by children, the instructions should be read by the experimenter. In the instructions, the test is referred to as a game.
- **Shape Game (five blocks).** In this part of the task, two response shapes appear below the center of the screen: a red circle on the lower left and a blue square on the lower right. On each trial, a red or blue target circle or square appears in the center of the screen, approximately twice as large as the response shapes. The participant must choose the response shape that matches the target in shape, but ignore the color. There are a total of five blocks of these trials, each of which is described in greater detail below.
- **Completion screen.** When the study is completed, a screen is shown that thanks the research participant. Text can be added here that debriefs participants about the hypotheses, goals, and outcomes of the study.

Administering the complete test takes under five minutes, including instructions and any debriefing. If more time is available and more statistical power is needed, longer trial blocks can be used.

#### Block types

In normal administration, the test consists of five consecutive test block types of the “Shape game”. Each block contains 20 trials by default (specified by the numtrials variable). Blocks include, in the order in which they are run:

- **Practice.** A block of six practice trials are given at the start of the task, with one example of each type of stimuli. On practice trials, audio feedback is given for correct (a pleasant beep) or incorrect (a buzzer) responses.
- **Neutral.** The neutral block consists of black outlines of circles and squares with no fill color to present distraction from the task of matching the shape.
- **Congruent.** The congruent block consists only of stimuli that match a single target in both shape and color. That is, only red circles and blue squares are used.
- **Incongruent.** The incongruent block consists of stimuli that match in shape but not in color. That is, only blue circles and red squares appear as targets. Instructions remain to match the shape.
- **Mixed.** The mixed block consists of all six targets, (congruent, incongruent and neutral). The mixed block contains half as many targets of each type as appeared in the earlier homogeneous blocks; by default 5 per type for a total of 30 trials.

#### Source code of the BST test package

The source code of the BST is organized to enable direct modification by users. The files included are listed in **Table 1.**

The BST is designed to be used as-is, so as to best match previous data collection. However, a number of aspects can be adapted to suit needs of experimenters, including image and sound files (see **Table 1**), and a set of control parameters (see **Table 2**). In addition, because the PEBL executable parses and compiles the source code of the BST, modifications can be made to the logic of the test by editing the source code directly. Instructions for writing and modifying PEBL code are available on-line (http://pebl.sourceforge.net/wiki/index.php/CogSci2011_Tutorial) and in the PEBL Manual [5].

#### Control parameters

**Table 2** identifies various parameters available for experimental control, their possible and default values, and what they control. In the initial release of the BST, these are all set near the beginning of the Start() function within the BST.pbl file. Version 0.14 of PEBL has functionality to set these parameters from the launcher itself, and their values can be edited by using the Set Parameters button in the launcher.

#### Input

Depending on parameters set by the experimenter, the response is made either by touching the response shape on a touchscreen interface, clicking the response shape using a mouse controller, or using the left and right shift keys on the keyboard. The default behavior is set to use keyboard input.

#### Data output

Three data files are saved in the data\ subdirectory of the BST directory. First, a .csv file is saved in the data\ subdirectory of the experiment (called BST-XX.csv, where XX indicates the participant code). This file records each trial for each participant on a separate line. A data record is made for each trial, with columns: subnum, type, block, congruency, trial, stim, resp, corr, rt, tooslow.

Here, subnum indicates a participant code entered by the experimenter, type indicates the block type (practice, neutral, congruent, incongruent, or mixed), block indicates the serial order of the block with 0 indicating the practice block, 2 the pure neutral block, 3 the pure congruent block, 4 the pure incongruent block, and 5 the mixed block. Congruency indicates whether for each particular trial, the irrelevant information (i.e., color) was congruent or incongruent with the correct response target. Neutral (black and white) stimuli are recorded as 0, stimuli that match shape and color (congruent) are recorded as 1, and stimuli that match shape but mismatch color (incongruent) are recorded as −1. Trial records a trial number (restarting from 1 on each block). Stim indicates the stimulus type, with 1= red circle; 2=red square; 3=blue square; 4=blue circle; 5=unfilled circle, 6=unfilled square. The resp column indicates whether the response was made to the left or right target, using the indicators <lshift> and <rshift>, regardless of the response modality. Corr indicates whether the response was correct (1 or 0), and rt indicates the response time, in ms.

**Figure 4:**
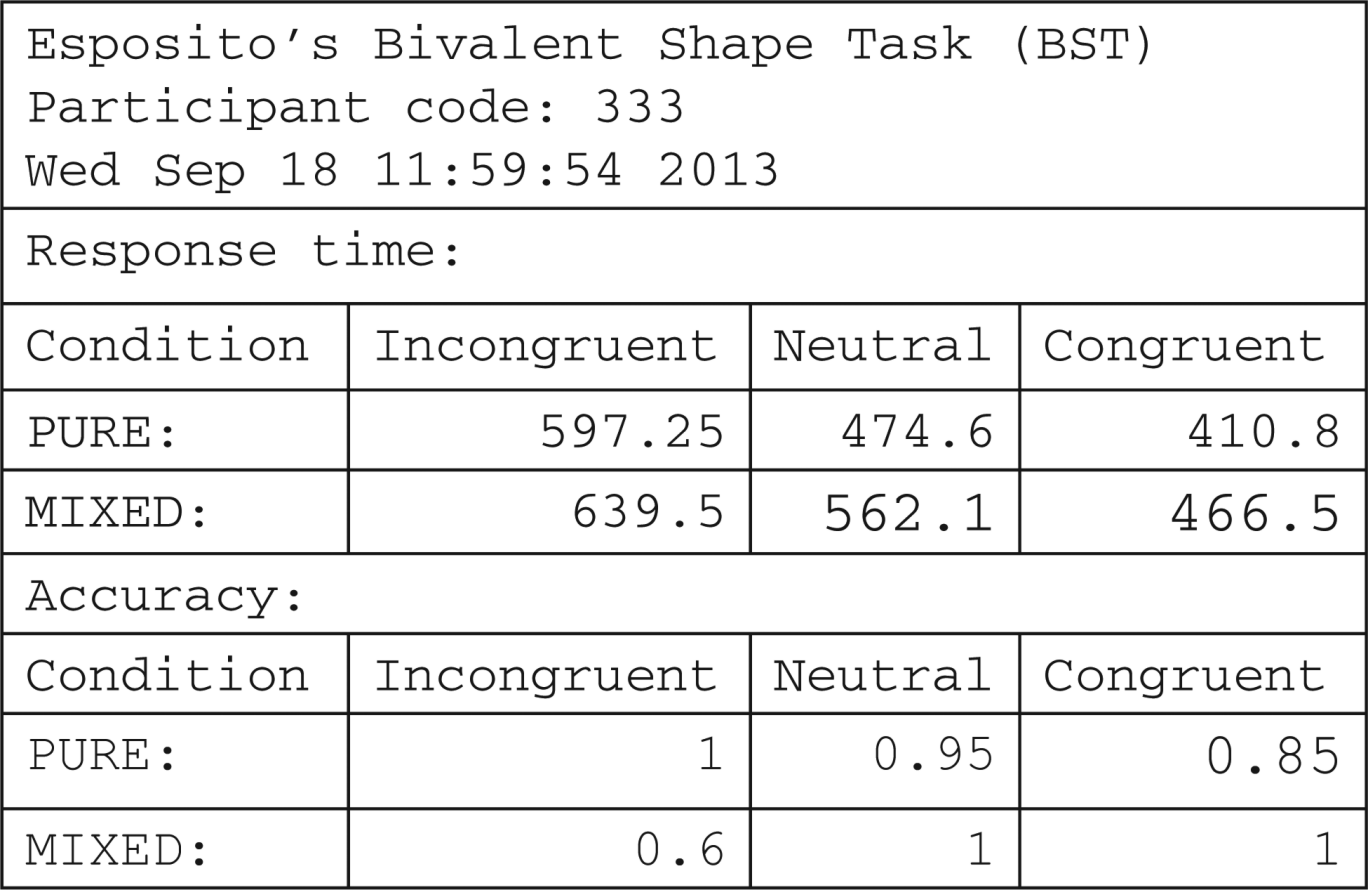
Example Report from the BST, showing mean response time (in ms) and accuracy for each of six conditions.

In addition, a second file (data\report-XX.txt) is created that summarizes the main results (mean response time and accuracy) across the primary conditions of interest in the study. An example of this file is shown in **Figure 4**. Primary dependent variables of interest include the response time costs and benefits of congruency (i.e., comparing congruent and incongruent response time to neutral), as well as how these are impacted by the mixed versus pure block manipulation.

Finally, these main results are recorded in a second file (data/pooled.csv), in comma-separated tabular format, so that each run of the study writes a single line to the file, to more readily enable group analysis and comparisons. Columns of this file are: participant code, timestamp, ACC_PI, ACC_PN, ACC_PC, ACC_MI, ACC_MN, ACC_MC, RT_PI, RT_PN, RT_PC, RT_MI,RT_MN, RT_MC.

Here, ACC indicates accuracy, RT indicates response time, P/M indicates pure versus mixed blocks, and C/N/I indicates congruent, neutral, and incongruent trials.

#### Response feedback

Audio feedback can be given after each response via pre-recorded .wav files. Correct responses use ‘beep.wav’ a pitch-ascending beep; incorrect trials use ‘buzz.wav’, an unpleasant buzzer, both of which can be changed or re-recorded by an experimenter. Visual feedback can be provided on each trial by setting the variable gUse-VisualFeedback from 0 to 1 (which will show ‘correct’ or ‘incorrect’ following each response). Auditory feedback can be provided on each trial by setting the variable gUseAudioFeedbackAlways from 0 to 1. Auditory feedback on only the first five trials of the practice blocks can be provided by setting the variable gUseAudioFeedbackPractice to 1 (the default) or 0 to turn this option off. By default, audio feedback is only given on the first five trials of practice blocks, and no visual feedback is given.

#### Repeatability

By default, the test seeds the random number generator with a common seed so that each testing session contains the identical set and order of stimuli. This order may not be identical across different computing platforms or versions of an Operating System, but are typically the same for similar computers. By setting gUseRandom to 1 instead of 0 in the source file, a new pseudo-random order of stimuli are chosen on each run, better enabling the test to be run multiple times on an individual.

### Quality control

In developing the test, we performed detailed testing across four sites, using multiple computers and operating systems (Several versions of Microsoft Windows, Apple's OS X, and Linux) to ensure the testing protocol worked as intended. We have verified that summary statistics produced by the program are identical to those computed on the raw data output, computed using external statistical software.

In addition, the software has been used in three distinct research studies at the NCSU Lifespan Developmental Psychology Laboratory, including: 1. a study with bilingual and monolingual preschool children completing the mixed block with only congruent and incongruent stimuli presented (74 participants; see [1,2]); 2. A study with 254 bilingual and monolingual early school age and late school age (K-1 and 4-5) children completing all blocks; and 3. A study with 100 college students where performance on a mixed block of congruent and incongruent stimuli was compared to performance on other inhibition tasks such as the color-word Stroop task (interference suppression) and a Go/No-Go task (response inhibition).

In general, PEBL has been shown to provide reliable and precise timing properties (see [3]), with response collection possible within 5 ms precision, presentation that can by synchronized to the screen update frequency, and timing precision that is typically within 1 ms of the desired timing.

## (2) Availability

### Operating system

The software will run using the PEBL Version 0.13, which is available for Linux, Apple's OS X (version 10.7 or later; 32- and 64-bit), and Microsoft Windows (Version XP or later; both 32- and 64-bit). The newest version of PEBL can be obtained for each operating system at http://sourceforge.net/projects/pebl/files/latest/download. Installing PEBL on a Debian-derived linux system (such as Ubuntu) can be done from the command line using the following commands:

> sudo dpkg -i pebl-0.13-linux-3.2-intel.deb
> sudo apt-get -f install


Once installed, the PEBL executable will be installed in / usr/bin/pebl, and running the BST.pbl executable is accomplished using the command > pebl BST.pbl from the directory where the BST archive has been decompressed.

### Programming language

The software was implemented on PEBL Version 0.13, which was released December 2012.

### Additional system requirements

The software can optionally be run using either keyboard input, mouse, or a touch-screen interface.

### Dependencies

The software requires PEBL Version 0.13 or later, which was released December 2012.

### List of contributors

- Shane T. Mueller (Implementation and Experimental Design; Michigan Technological University)
- Alena Esposito (Primary Experimental Design; NCSU)

### Software location

The BST code is available via the PEBL Project sourceforge website as part of a larger library of psychological tests, as well as a github repository for the BST task.

### Archive

sourceforge.net

#### Name

The PEBL Sourceforge project site.

#### Persistent identifier

http://sourceforge.net/projects/pebl/files/special/BST-1.1.zip/download

#### License

GPL Version 3 (GPLv3)

#### Publisher

Shane T. Mueller

#### Date published

14/03/2014

### Code repository

Github

#### Name

The BST task Github repository Version 1.1.

#### Identifier

https://github.com/stmueller/PEBL_BST

#### License

GPL Version 3 (GPLv3)

#### Date published

14/03/2014

#### Support

Support for installing and running PEBL and the BST are available via the PEBL email list (pebl-list@lists.source-forge.net), or via the PEBL discussion forums (http://sourceforge.net/p/pebl/discussion/). Additional support material can be obtained via the PEBL wiki (http://pebl.sourceforge.net/wiki/index.php), a periodic series of blog posts (http://peblblog.blogspot.com), and a repository of supplemental documents (http://figshare.com/articles/PEBL_Support_Materials/961767).

### Language

The BST is programmed using the Psychology Experiment Building Language (PEBL), a special-purpose language designed for implementing computerized behavioral testing.

## (3) Reuse potential

The BST joins a growing number of executive function tasks available within the PEBL test battery (including versions of [7-10]; [3] identifies approximately 70 computerized psychological tasks available within the PEBL Test Battery). These also included tests targeted toward and validated with child samples [11,12]. One advantage of having such a large repository of tests is that it enables a battery of tests to be run in sequence using a single testing platform, with data tied together to enable comparison for reliability and validity studies. This is also facilitated by the fact that the BST is a relatively short task, taking under 5 minutes to complete.

The task is easily modifiable to suit new purposes. Even without developing new software code, imagery and sounds can be altered, and a number of parameters offer control of which trial blocks are used, how many trials are tested, the amount of time permitted for a response, and the text instructions and feedback. This allows future researchers to tailor the task to their particular needs and age group without running into floor or ceiling effects. Furthermore, the task could easily be modified to use additional stimulus-response block types not available in the original test.

We believe the BST may have potential as a brief test of executive function and attentional filtering in many contexts, including as a means of judging neurological integrity, attentional deficits, impulsivity, the psychological impact of stressors, developmental improvement and age-related decline. Future behavioral research must be done to establish the task's validity in these and other contexts. 

## Figures and Tables

**Fig. 3:**
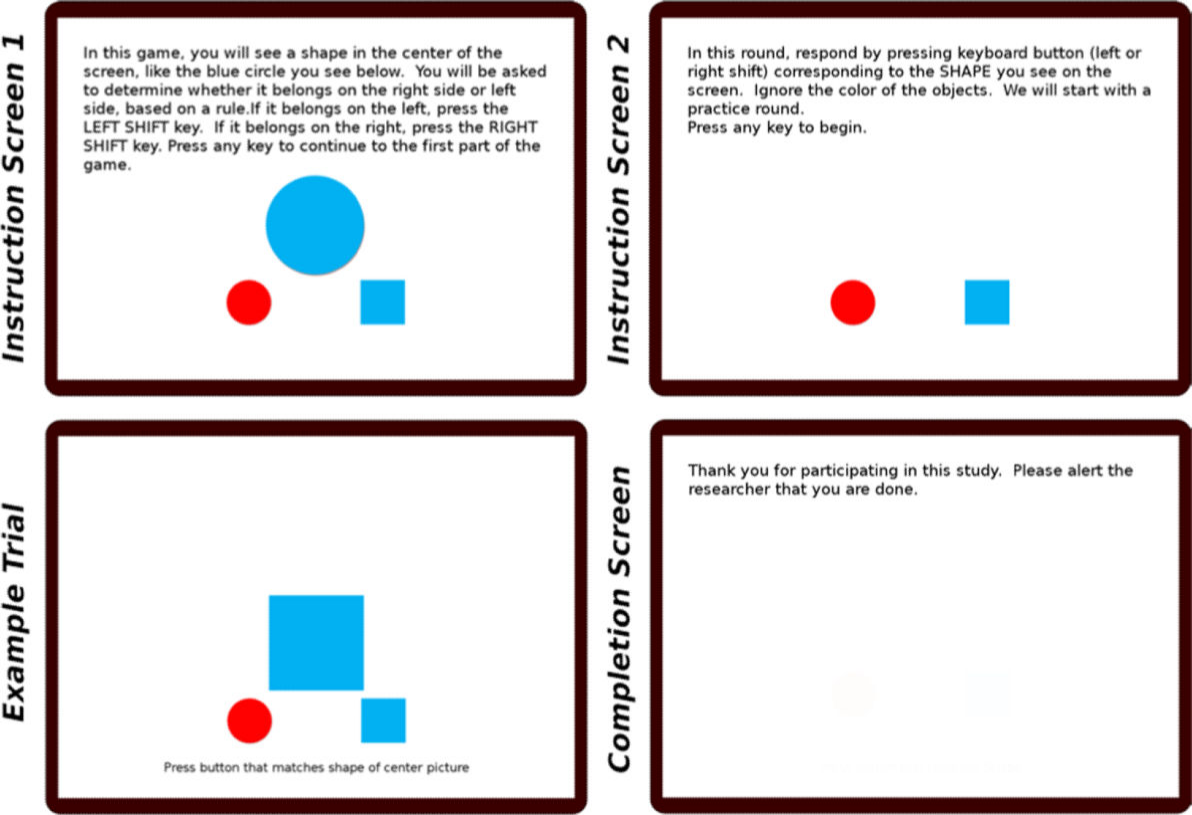
Example instruction, stimulus, and completion screens of the Bivalent Shape Task (BST).

**Table 1:** Files included in the BST package. Image and sound files can be replaced to adapt the task for other purposes.

Filename	Description
BST.pbl	PEBL source code file
LICENSE	Copy of GPLv3
BST.pbl.png	Screenshot of test for launcher
README.md	Description of test
blackcircle.png	Stimulus image
blacksquare.png	Stimulus image
bluecircle.png	Stimulus image
bluesquare.png	Stimulus image
redcircle.png	Stimulus image
redsquare.png	Stimulus image
beep.wav	Auditory feedback for correct trials
buzz.wav	Auditory feedback for correct trials

**Table 2:** Variables that control basic experimental properties.

Variable	Values	Impact
gUseRandom	0 (not random) or 1 (random)	Each run uses either an identical (0) or unique (1) order.
gResponseOffset	100	Left/right offset from center of response targets.
gResponseY	screenheight/2+150 (default)	Distance, in pixels, from top of screen, of response targets.
gResponseTimeLimit	3000 (default)	How long (in ms) a response must be before trial is aborted.
gUseVisualFeedback	0 (default) or 1	Always use visual feedback.
gUseAudioFeedbackAlways	0 (default) or 1	Always use auditory feedback.
gUseAudioFeedbackPractice	0 or 1 (default)	Use auditory feedback during practice trials.
numtrials	default is 20	Baseline number of trials per block.
gUseMouse	0 (default; keyboard) 1	Determines whether mouse or keyboard should be used for response collection and instructions.
